# Spontaneous bilateral distal ulna fracture: an unusual complication in a rheumatoid patient

**DOI:** 10.1007/s10195-011-0140-4

**Published:** 2011-05-20

**Authors:** Santosh Venkatachalam, Paul Dixon

**Affiliations:** 1 Department of Orthopaedics, Sunderland Royal Hospital, Kayll Road, Sunderland, SR4 7TP UK; 2 67 Greenlee Drive, Newcastle upon Tyne, NE7 7GA UK

**Keywords:** Bilateral, Ulna, Stress fractures, Rheumatoid arthritis

## Abstract

Bilateral ulna stress fractures are extremely rare. Patients with rheumatoid arthritis have osteopenic bone secondary to a variety of causes. We report a case of bilateral stress fractures of the ulna in an elderly patient with rheumatoid arthritis, and literature on this condition is reviewed. Prompt recognition and activity modification are essential to treat this rare injury. Recovery can take up to 12 weeks.

## Introduction

Cyclical loading with the failure of bone to adapt to its mechanical environment leads to stress fracture. They are more common in lower limb weight-bearing bones [1, 2]. There have been many reports of stress fractures of the ulna in relation to sporting activities [3–7]. Stress fractures are an unusual but increasingly recognised complication of rheumatoid disease and its treatment with a prevalence of 0.8% in patients with rheumatological illness [8].

They are commonly mistaken for other joint conditions, like infection or exacerbation of inflammatory arthropathy [9]. Despite a frequent delay in diagnosis and sometimes further unnecessary investigations due to a lack of knowledge, they carry a good prognosis with conservative management in these patients [10, 11].

To our knowledge, this is the first case of bilateral stress fractures of the ulna to be reported in rheumatoid arthritis. A higher index of clinical suspicion is required for early diagnosis and to institute appropriate treatment/rehabilitation.

## Case report

A 71 year old lady with a 6-year history of seropositive rheumatoid arthritis presented to her general practitioner with bilateral swelling and pain in the forearm.

She had been on methotrexate, which was stopped a few weeks prior to presentation because of breathlessness secondary to rheumatoid and methotrexate-induced pneumonitis. She was put on a short course of high-dose prednisolone, 30 mg/day, for her pneumonitis. Other medications included etodolac 600 mg daily, paracetamol for analgesia and lansoprazole 15 mg daily.

She developed swelling in the right forearm a few weeks after being on prednisolone, followed by similar swelling in the left forearm a couple of weeks later. When telephonic advice was sought from the rheumatologists, they did not suspect a fracture as there was no history of trauma. As she was already on steroids, they felt that it was unlikely to be an exacerbation of her rheumatoid arthritis. Hence, she was asked to keep her normal follow-up clinical appointment to see them. In view of her persisting symptoms, radiographs were organised by her general practitioner and she was then referred to the fracture clinic for further opinion.

**Fig. 1:**
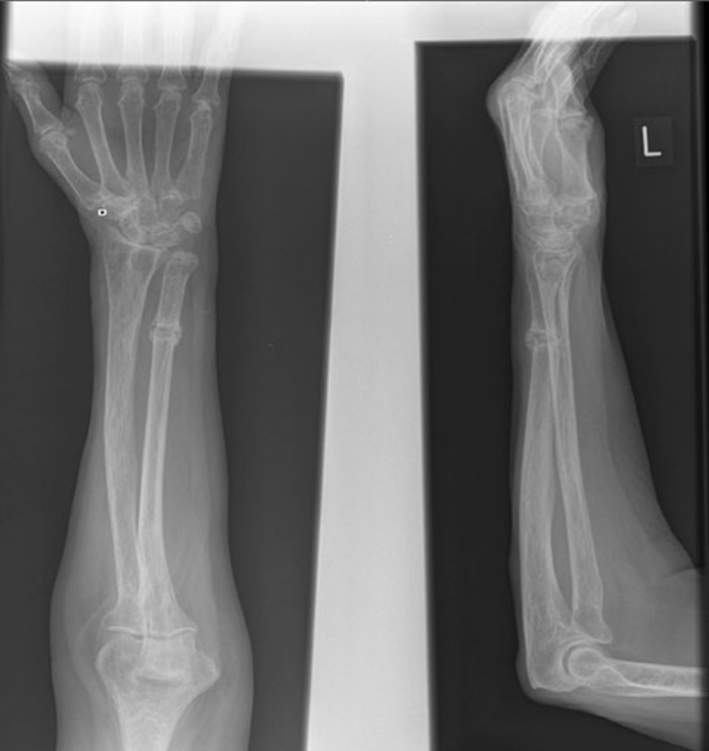
Left ulna fracture

**Fig. 2:**
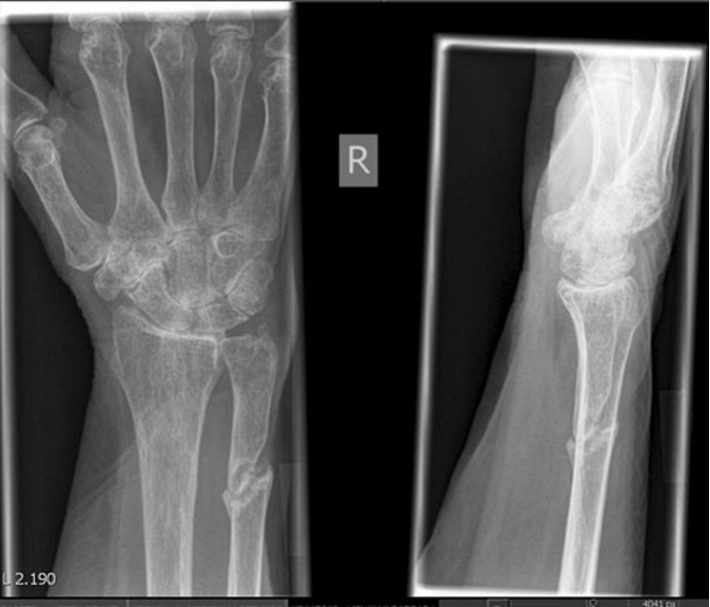
Right ulna fracture

On direct questioning, she gave a history of having recently moved to her daughter’s house. She had to use her hands to go up the stairs holding on to the banister as she also had right knee arthritis for which she was awaiting total knee replacement. Clinical examination revealed tenderness over the distal one-third of both ulnae with no obvious deformity and an intact distal neurovascular status. Wrist examination did not reveal any synovitis/effusion/exacerbation of arthritis or infection. Active flexion/extension of the elbow and pronation/supination of the forearm were restricted terminally due to pain. Assessment of radiographs confirmed bilateral short oblique fractures of the distal one-third of the ulna with intact distal radioulnar joints (Figs. 1, 2). The bones appeared osteopenic with some arthritic changes in the distal radioulnar joint and wrist joint.

Her DEXA scan suggested a lumbar spine T score of −1.5 (Z score of −0.4), a total hip (left) T score of −1.5 (Z score −0.1), and a right hip T score of −2.1 (Z score −0.8), suggesting osteopenia.

The fractures were treated nonoperatively with elastic tubular bandage. The patient was advised to avoid pulling herself up the banister with her forearms. Occupational rehabilitation therapy was organised, and changes were made to her home setup so that she could negotiate stairs with a stair lift instead of using her arms. Both fractures healed well with conservative management by 12 weeks.

The patient gave informed consent prior to the submission of this case report.

## Discussion

Spontaneous fractures of the iliac blade [1], metatarsals, lower tibia and fibula [2], the femoral neck and pelvic bones have been reported in rheumatoid disease. Stress fractures of the ulna and radius are rare. The middle third of the ulna is the commonest site, as this region has the thinnest cortex and smallest cross-sectional area morphologically compared to the proximal or the distal third, and is vulnerable to stress from torsional forces [12].

Fractures of the distal one-third of the ulna are commonly described as night stick transverse fractures following direct trauma. Unilateral ulna stress fractures have been reported in softball pitchers/volleyball players [3], tennis players who use a double-handed backhand stroke [4], a golfer [5], a baseball player [6] and a ten pin bowler [7].

Plain radiographs are performed as a first line investigation. Radiographs have low sensitivity (15%) but high specificity (50%), and a stress fracture may be identified by new periosteal bone formation, sclerosis, callus or a clear fracture line, but can take up to 3 months [13]. Bone scan has a sensitivity of 100%, but is not specific as a hot spot may be caused by nontraumatic conditions like osteoid osteoma, osteomyelitis or bone infarct [14]. Computed tomography (CT) can be useful to differentiate between these conditions [15]. Magnetic resonance imaging (MRI) has a high sensitivity similar to that of bone scan, with the added advantage of excellent anatomic visualisation, and is being advocated as the investigation of choice [16].

Bilateral stress fractures of the ulna are very rare and have been reported on two occasions in adolescent patients in the literature. In the paper by Sujino et al. [17], a 15 year old adolescent male Japanese fencing player developed bilateral stress fractures of the ulna following his first training camp. In this type of fencing activity, the player grasps the special sword, which weighs about 500 g and is 115 cm long with both hands, and swings it up and down repeatedly. Stress fractures of both ulnae have been reported secondary to using crutches [17–19]. Steunbrink et al. [20] reported the first bilateral stress fractures of the ulna in an adult in a weightlifter secondary to a bowing mechanism caused by the biceps and flexor muscles that generates a supination–flexion movement of the ulna, leading to recurrent microtrauma.

This is the first case of bilateral ulna stress fractures reported in a patient with rheumatoid arthritis. We were unable to identify a similar case in a review of literature from around the world. Predisposing factors in this lady include her rheumatoid arthritis, osteoporosis and steroid intake. She was using the forearms to pull herself up the stairs holding on to the banister. It is likely that the cause of fracture in this case was a combination of torsional and tractional forces caused by cyclical weight bearing due to holding on to the banister, with the wrist in a position of flexion, ulnar deviation and pronation resulting in increased axial load through the ulna, leading to fracture [16, 21, 22]. She was not using any devices for ambulation like crutches, and did not have any history of definite trauma for the doctor to suspect a stress fracture. The bilaterality and location of the fractures close to the wrist easily misled the examining clinician to suspect exacerbation of her rheumatoid arthritis or infection in the wrist joint rather than a fracture.

Diagnosis of a stress fracture requires a detailed history, a precise musculoskeletal examination, clinical experience and adequate knowledge. The local redness, swelling and warmth near a joint in a patient with pre-existing rheumatoid arthritis taking steroids is often misinterpreted as either a relapse of the disease in the joint or the development of an infection like septic arthritis/cellulitis. Since the painful symptoms overlap with the clinical picture of painful joint diseases, and because of the low sensitivity of conventional diagnostic X-rays, insufficiency fractures are not diagnosed directly or their diagnosis is delayed. Spontaneous/exercise-related pain in the extremities in association with tenderness localized over the bone rather than the joint, particularly in the absence of other signs of rheumatoid activity, should make the clinician suspect a stress fracture.

This case highlights the need for the treating physician/surgeon/radiologist to be aware of these injuries in patients with rheumatoid arthritis. Patients, especially those on steroids, need to be educated about the risks of stress fractures. Patient education, appropriate measures such as activity modification, occupational therapy and treatment for osteoporosis should be instituted to prevent such morbidities.

Stress fractures of the upper limb are infrequent but not rare. The treating physician should consider stress fracture as a possible diagnosis in cases of upper limb pain of bony origin with no acute joint pathological findings where the pain is associated with overuse or in patients with an associated pathology. Imaging such as plain radiographs, bone scan and computed tomography/magnetic resonance imaging can be used to confirm the diagnosis.
